# Identification of Key Aroma Compounds in Type I Sourdough-Based Chinese Steamed Bread: Application of Untargeted Metabolomics Analysisp

**DOI:** 10.3390/ijms20040818

**Published:** 2019-02-14

**Authors:** Bowen Yan, Faizan A. Sadiq, Yijie Cai, Daming Fan, Hao Zhang, Jianxin Zhao, Wei Chen

**Affiliations:** 1 State Key Laboratory of Food Science and Technology, Jiangnan University, Wuxi 214122, China; yanbowen2011@foxmail.com (B.Y.); faizan_nri@yahoo.co.uk (F.A.S.); fandm@jiangnan.edu.cn (D.F.); zhanghao@jiangnan.edu.cn (H.Z.); chenwei66@jiangnan.edu.cn (W.C.); 2 School of Food Science and Technology, Jiangnan University, Wuxi 214122, China; 3 Suzhou Tourism and Finance Institute., Suzhou 215104, China; jiangnan_caiyijie@foxmail.com; 4 National Engineering Research Center for Functional Food, Jiangnan University, Wuxi 214122, China; 5 Beijing Innovation Centre of Food Nutrition and Human Health, Beijing Technology and Business University (BTBU), Beijing 100048, China

**Keywords:** Chinese steamed bread, type I sourdough, metabolomics, odor activity value, aroma compounds

## Abstract

Untargeted metabolomics is a valuable tool to analyze metabolite profiles or aroma fingerprints of different food products. However, less attention has been paid to determining the aroma characteristics of Chinese steamed breads (CSBs) by using this approach. The aim of this work was to evaluate the key aroma compounds and their potential generation pathway in Chinese steamed bread produced with type I sourdough by a metabolomics approach. Based on the aroma characteristics analysis, CSBs produced with type I sourdough and baker’s yeast were clearly distinguishable by principal component analysis (PCA) scores plot. A total of 13 compounds in sourdough-based steamed breads were given the status of discriminant markers through the untargeted metabolomics analysis. According to the odor activity values (OAVs) of discriminant aroma markers, ethyl acetate (fruity), ethyl lactate (caramel-like), hexyl acetate (fruity), (E)-2-nonenal (fatty) and 2-pentylfuran (fruity) were validated as the key volatile compounds in the breads produced with type I sourdough as compared to the baker’s yeast leavened steamed bread. The metabolite analysis in proofed dough indicated that esters are mainly generated by the reaction between acid and alcohol during steaming, and aldehydes are derived from the oxidation of palmitoleic acid and linoleic acid during proofing and steaming.

## 1. Introduction

The aroma of Chinese steamed bread (CSB) is one of the most important sensory attributes that determines the quality of the bread and consumers’ acceptability [1]. Despite the ease of using baker’s yeast in breads, type I sourdough is still preferred because of a plethora of benefits it confers to the bread. For instance, the complex microbiota of type I sourdough not only improves the flavor (aroma and taste) [2] texture [3] and shelf life [4], but also enhances the nutritional/functional characteristics of cereal-based foods [5]. In type I sourdough, microorganisms are kept metabolically active by daily back-slopping, and bread leavening is sustained by microbiota without the need of adding baker’s yeast [6]. The role of microbes in conferring aromatic properties to fermented cereal products is well-known, and a strong correlation between representative microbiota of type I sourdough and volatile compounds was previously reported by us and other researchers [1,7].

Due to the importance of aroma characteristics in reaching consumer expectations, high marketability and product development of breads, sensory and instrumental evaluations have been widely used to determine the aroma profile of many types of breads [8,9]. However, there is less focus on the aroma characteristics of CSBs despite the fact that there is a significant variation in the microbiota of sourdoughs which have an ultimate influence on the aroma profile. The key aroma compounds of CSB made with sourdough were also revealed by using three different aroma extraction methods (solid-phase microextraction, simultaneous distillation-extraction and purge and trap) to compare the relative peak area of volatiles among six samples [10]. However, only a small proportion of volatile compounds that contribute to the CSB aroma, depending on the content and the perception threshold, was reported [11]. It is noteworthy that the total food aroma may comprise hundreds of compounds, all of which are not equally important or responsible for shaping the aroma of a product, and thus screening potential discriminant compounds of a product is indispensable to explore potential aromatic volatiles. Untargeted metabolomics has frequently been used in clinical research as well as in food systems for the identification of characteristic aroma compounds of a product or to determine discriminant volatiles [12,13,14]. Although specific aroma compounds with higher concentration or odor activity values (OAVs) have been reported and used to evaluate the aroma feature of bread or CSB, these compounds are limited to overall representation of the CSB aroma property. Therefore, the combination of the untargeted metabolomics analysis and odor threshold techniques could potentially provide a better and comprehensive overview of the key aroma compounds of a CSB produced with different starter cultures.

The complex microbiota of sourdough may have a great influence on the volatiles of the final product through the generation of different volatile precursors after the proofing step (i.e., precursors of lipid oxidation and the Maillard reaction) [15]. The precursors of volatile compounds in CSB are derived from enzymatic and microbial processes during sourdough fermentation, but less attention is paid to the content of precursors during sourdough fermentation. Thus, the effect of sourdough addition on the precursors during fermentation and their aroma compounds in CSB remains unclear.

Therefore, the first objective of this study was to evaluate the key aroma compounds in Chinese steamed bread produced with type I sourdough through the combination of the untargeted metabolomics analysis and odor threshold techniques. The second objective was to provide insights into the relationship between the precursors in the dough after proofing with type I sourdough and the generation of key aroma compounds in the corresponding CSBs.

## 2. Results

### 2.1. Aroma Feature Assessment of CSB by E-Nose

**Figure 1:**
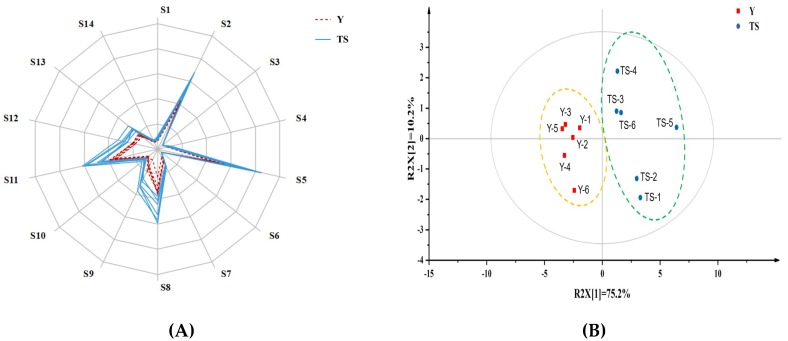
Response radar plot (**A**) and principal component analysis (PCA) scores plot (**B**) of aroma signals of CSB produced with baker’s yeast (Y) and type I sourdough (TS), respectively. S1-S14 represented 14 different individual metal oxide semiconductors (MOS) in the e-nose.

Electronic nose (e-nose) as a non-destructive and objective method was used to investigate the aroma profile of CSBs made with type I sourdough and baker’s yeast. Radar charts illustrated differences in the aroma profile of different CSBs based on metal-oxide semiconductor (MOS) gas sensor (Figure 1A). The signal values of sensors S2, S5, S8 and S11 were relatively higher than the signal values of other sensors, which indicated that these four sensors were highly sensitive to the aroma of CSB samples. Sensors S5 and S11 of the e-nose were sensitive to esters and aldehydes among all aromatic compounds. Previous reports on the aroma of CSB found that esters and aldehydes were proved to the main compounds in sourdough for contributing to the aroma of products [16]. Based on the e-nose data, responses from all MOS sensors for CSB samples were subjected to PCA (Figure 1B). The first two principal components accounted for 85.4% of the total variance for samples. The samples of different groups were clearly distinguished by PC1 into two clusters, which suggested that CSB produced with type I sourdough resulted in the majority of the variance in the volatile composition compared to the corresponding reference samples fermented by baker’s yeast. The e-nose data confirmed that there was a more pronounced effect of type I sourdough on the aroma properties of the CSB samples.

### 2.2. Effect of Type I Sourdough on the Volatile Compounds of CSB

**Figure 2:**
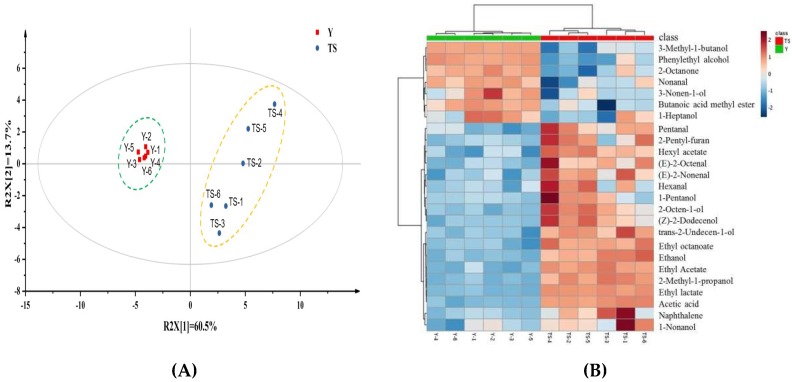
Principal component analysis (PCA) score plot (**A**) and heatmap analysis (**B**) of the headspace volatiles identified in Chinese steamed bread produced with baker’s yeast (Y) and type I sourdough (TS).

Solid-phase microextraction and gas chromatography coupled to mass spectrometry (SPME-GC/MS) has been demonstrated to be an effective method for the aroma analysis of bread or steamed breads [17,18]. In our study, volatile compounds in wheat bread samples fermented by type I sourdough and yeast were determined after proofing and steaming by using SPME-GC/MS. In the results shown in supplementary Table S1, a total of 34 compounds, including 15 alcohols, 8 aldehydes, 6 esters, 3 ketones, 1 acid, and 1 furan were found in all the samples. The presence of these compounds was verified by comparing different mass spectral and retention index (RI) libraries. The principal component analysis (PCA), based on volatile compound contents in all products, showed that CSBs produced with type I sourdough and yeasts are markedly different in terms of their volatile compounds and thus clustered separately (Figure 2A). The results of the SPME-GC/MS analysis were in line with the results obtained using the e-nose in our study. Heatmaps were constructed to show differences in the concentration of major volatile compounds between the CSBs made with two different starters (Figure 2B). It is clear that CSBs produced with type I sourdough could be distinguished from the yeast-based breads due to the abundance of aldehydes, esters and acetic acid contents, with the exception of nonanal and butanoic acid methyl ester.

### 2.3. Identification of Key Volatile Compounds for CSB Produced with Type I Sourdough

**Figure 3:**
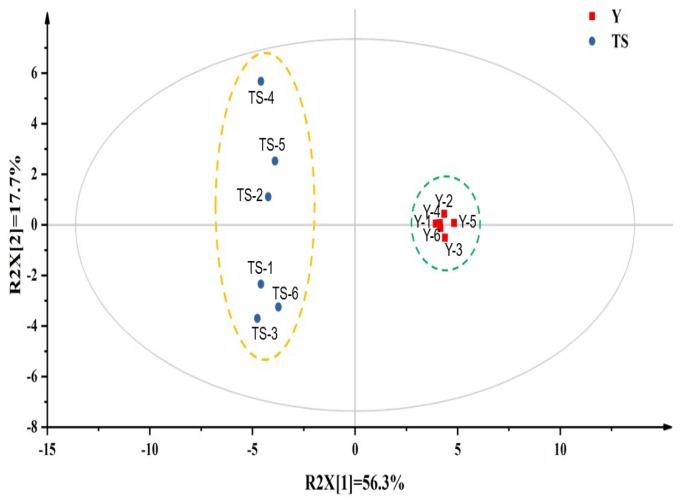
Partial least-squares-discriminant analysis (PLS-DA) scores plot of the headspace volatiles identified in Chinese steamed bread produced with baker’s yeast (Y) and type I sourdough (TS).

To further explore the key volatile compounds responsible for the aroma profile of the CSB produced with type I sourdough, an untargeted metabolomics approach was used. The PCA clearly distinguished CSBs, produced by different starter cultures, based on the concentration of aroma compounds. Subsequently, the partial least-squares-discriminant analysis (PLS-DA) model was applied for the cross-validation of the PCA results and to determine the key compounds responsible for the discrimination of two groups of CSBs. The PLS-DA scores plot revealed that samples of two groups were clearly distinguishable, with the model parameters R2Y = 0.994, R2X = 0.740, and Q2 = 0.970 (Figure 3). In total, 100 permutations of the rows were performed, and the Q2 value (-0.439) certified the stability and reliability of PLS-DA model (Figure S1). According to the PLS-DA model, 13 compounds were selected as discriminant volatile compounds of the samples produced with type I sourdough that made them markedly distinct from the other group (Table 1, *p* < 0.05, variable importance in the projection (VIP) > 1.0). The average concentration of acetic acid in the type I sourdough-based CSBs was 356 times greater than that in the control group, which may be the reason for the sourness of the CSBs fermented by type I sourdough. Moreover, the ethyl lactate, ethyl acetate, hexyl acetate and ethyl octanoate concentrations, which provide fruity aroma to CSBs, were 231, 117, 11 and 3 times higher, respectively, in samples made with type I sourdough as compared to the one made with baker’s yeast. In addition, some aldehydes (E-2-nonenal, pentanal, E-2-octenal and hexanal) and one furan (2-pentylfuran) were also determined at around 2 to 3 times higher concentration in CSB produced with type I sourdough. These aforementioned volatile compounds are the most commonly reported volatile compound in fermented foods due to their low odor threshold [19,20,21]. It is worth noting that only a small proportion of volatile compounds have an impact on the CSB aroma depending on the concentration and the perception threshold of volatile compounds. In this study, we combined odor threshold techniques and untargeted metabolomics analysis to characterize the key aroma compounds in CSB produced with type I sourdough. The values of odor threshold in water are summarized in Table 1 as previously suggested by Leffingwell et al. [22] (online), followed by the odor activity values (OAVs). The OAVs determine the potency of odorants in food and allow researchers to differentiate between odorless volatiles and aroma compounds which truly contribute to the aroma feature of food. If the OAV of a volatile compound is lower than 1.0, it indicates there is no contribution of the compound to the aroma of the products [23]. Although CSBs produced with type I sourdough had very high concentrations of ethanol and acetic acid, the OAV of alcohols, acids and pentanal from the list of discriminating compounds (Table 1) were < 1 in both types of breads. Similarly, the concentration of esters in yeast-based CSBs was lower than their odor threshold. Notably, based on the OAVs, ester compounds (ethyl lactate, ethyl acetate, hexyl acetate) had a high contribution towards the aroma of CSBs produced with type I sourdough. Moreover, (E)-2-nonenal, (E)-2-octenal, hexanal and 2-pentyl furan in CSB samples fermented by both starter cultures had an OAV >1. The OAVs of aldehydes and 2-pentyl furan were about 2 and 3 times higher, respectively, in the bread samples fermented by type I sourdough than those fermented by the yeast. Using the untargeted metabolomics approach and odor thresholds of aroma compounds, we concluded that ethyl acetate, ethyl lactate, hexyl acetate, (E)-2-nonenal and 2-pentylfuran, which had high VIP value and OAV, were the key volatile compounds of CSB produced with type I sourdough as compared to yeast-based breads. 

### 2.4. Potential Precursors in Proofed Dough for the Production of Volatile Compounds in Type I Sourdough-Based CSB 

**Figure 4:**
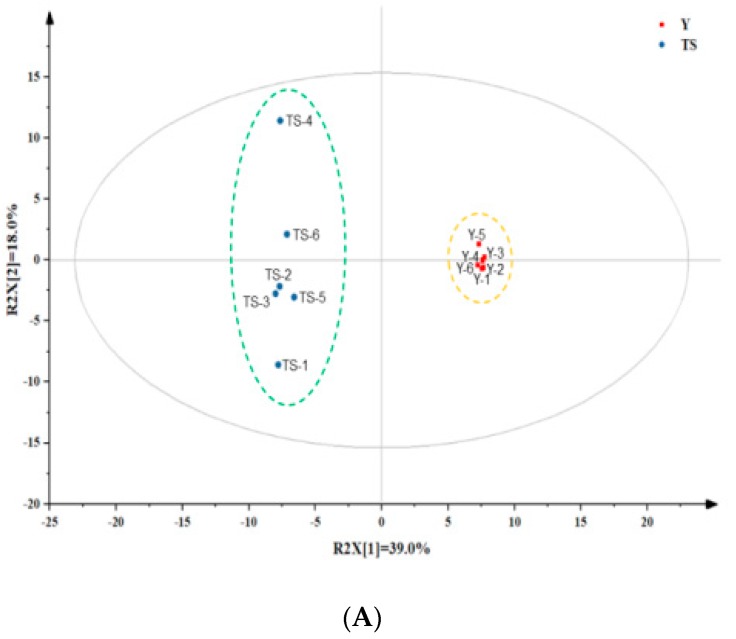

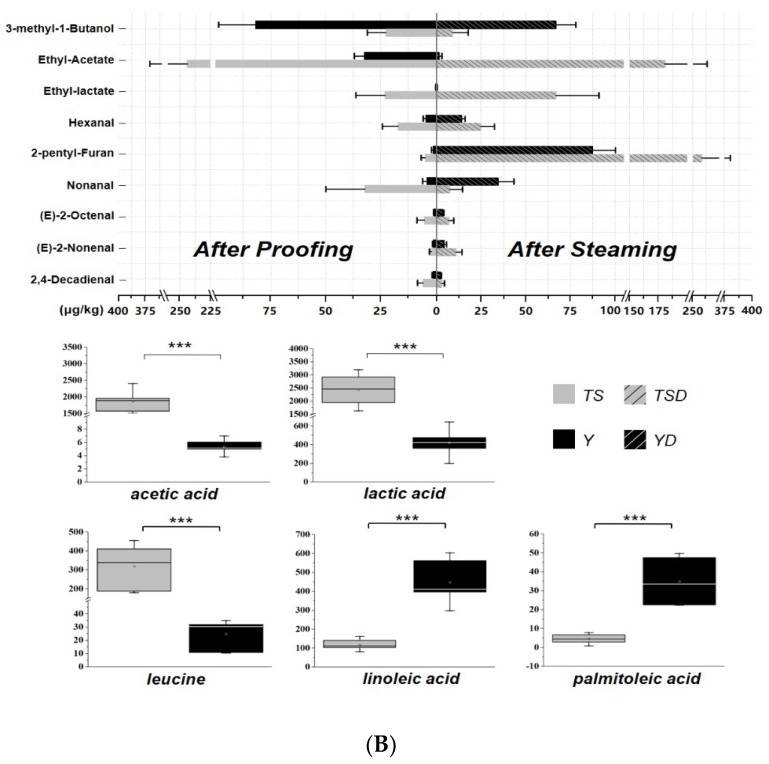
(**A**) Partial least-squares-discriminant analysis (PLS-DA) score plots of the potential metabolite markers in proofed dough fermented with type I sourdough (TS) and baker’s yeast (Y); (**B**) The concentration of characteristic volatile compounds in CSBs produced with type I sourdough and baker’s yeast and the specific precursor contents in the dough fermented with type I sourdough and baker’s yeast after proofing. All the results were performed in triplicate, and *** means *p* < 0.001.

Using the untargeted metabolomics analysis, the metabolites in proofed dough were identified by gas chromatography / time-of-flight mass spectrometers (GC-TOFMS) to explore the relationship between the precursor metabolites of the proofed bread and the key volatile compounds of CSB produced with type I sourdough after the steaming process. The first two principal components of the PCA plot clearly separated samples belonging to two different groups (Figure S2A). Subsequently, the PLS-DA model also divided the samples belonging to two groups into two clusters, with the model parameters R2Y = 0.998, R2X = 0.721, and Q2 = 0.975 (Figure 4A). The permutation results (*n* = 100; Q2 = −0.418) validated the stability and reliability of the PLS-DA model (Figure S2B). The S-plot (Figure S3) shows the distribution of metabolites: the metabolites far away from the origin represent potential discriminant compounds. A total of 41 potential markers with p values less than 0.05 and VIP values higher than 1.0 are summarized in supplementary Table S2. A total of 11 discriminant metabolites of the proofed dough were further selected (Table 2), including 5 acids, 4 amino acids and 2 unsaturated fatty acids that could possibly have a role in the generation of key aroma volatiles in the CSBs produced with type I sourdough From our results, higher concentrations of lactic acid and acetic acid produced by LAB were also found in the proofed dough fermented with type I sourdough, which is the driving force behind the pH drop in sourdough-based products. Leucine, histidine, asparagine and glutamic acid were also selected as important discriminant precursors for volatile compounds generation, which had higher concentration in the proofed dough fermented with type I sourdough. As shown in Figure 4B, unsaturated fatty acids (palmitoleic acid and linoleic acid) in the proofed dough fermented with type I sourdough were lower as compared to the dough fermented by yeast, but higher contents of hexanal, nonanal, (E)-2-octenal, (E)-2-nonenal, 2,4-decadienal and 2-pentyl furan were generated in the corresponding proofed dough, and their concentration were further increased during steaming expect for nonanal.

## 3. Discussion

Aroma properties of a bread depend on naturally present odorants in the flour [24], and interaction between the microbial metabolism and indigenous cereal enzymes during the fermentation process that leads to the synthesis of volatiles and their precursors [25]. Microbial fermentation is considered the most important route for the production of volatile compounds in sourdoughs. Differences in metabolic and kinetic ways of lactic acid bacteria (LAB) and yeast lead to the generation of specific volatiles during fermentation. Homofermentative and heterofermentative LAB also have different metabolic pathways that lead to the formation of different compounds. Acetic acid (vinegar) is the major volatile compound resulting from heterofermentative LAB fermentation and was proved to improve the aroma of bread with appropriate concentration, but has a negative effect in excessive concentration [26]. Ethyl acetate (fruity), ethyl lactate (caramel-like) and ethyl octanoate (fruity) are also specific to heterofermentative LAB [27], and these compounds were found in high concentrations in the type I sourdough-based CSBs as compared to the control. Similar to bread, lipid oxidation mainly leads to the synthesis of 2-pentylfuran (fruity) as well as aldehydes in the CSB, such as hexanal (green), nonanal (aldehydic), (E)-2-octenal (fresh), (E)-2-nonenal (fatty), benzaldehyde (almond-like) and 2,4-decadienal [28]. The compounds, produced as a result of lipid oxidation, may further be converted into their corresponding alcohols through the metabolic activities of heterofermentative LAB, such as (E,E)-2,4-decadienal and (E)-2-nonenal [29]. The bread fermented by baker’s yeast contains high concentration of phenylethyl alcohol, which has a typical rose-like odor and is derived from phenylalanine present in flour. Likewise, 3-methyl-1-butanol (fruity) is one of the most frequently reported compounds in bread or steamed bread where it exerts a positive influence on the overall aroma profile of the products [30]. Liu et al. [1] also reported the presence of high concentrations of 3-methyl-1-butanol, phenylethyl alcohol and 2-octanone in CSB fermented by *Saccharomyces cerevisiae*, which is the main species in baker’s yeast. 

As compared to yeast-based breads, ethyl acetate, ethyl lactate, hexyl acetate, (E)-2-nonenal and 2-pentylfuran were confirmed as the key volatile compounds of CSB produced with type I sourdough based on the odor threshold techniques and untargeted metabolomics analysis. Kaseleht et al. [27] described ethyl acetate, ethyl lactate and ethyl hexanoate as the main volatile compounds in sourdough, which indicated that type I sourdough had a great impact on ester contents of CSB. Ethyl acetate with a high concentration in all the samples was also found in the proofed dough and decreased after steaming in CSBs in our study. As previously reported by Birch et al. [31], esters are derived from fatty acids pathway in the yeast cell during fermentation. However, the other esters (ethyl lactate and hexyl acetate) were mainly generated in all samples by the reaction between acids and alcohols during the steaming process since the heating acts as a catalyst for the esterification reaction through comparison with the concentration of esters between the proofed dough and CSBs [32]. In addition, (E)-2-nonenal and 2-pentylfuran have been proved as the most common aroma-active compounds in wheat-based bread crumbs [21,31]. As shown by the results in supplementary Table S1, 2-pentylfuran is mainly generated during steaming since it was found in low concentrations in all the dough samples after proofing, which is in line with the finding of Birch et al. [31]. 

According to discriminant precursor analysis, lactic acid and acetic acid are also important precursors for the generation of ethyl acetate and ethyl lactate in the esterification reaction during steaming. For instance, ethyl acetate is produced as a result of the Fischer esterification reaction between acetic acid and ethanol [33]. Leucine, histidine, asparagine and glutamic acid were also selected as important discriminant precursors for volatile compound generation, which had higher concentration in the proofed dough fermented with type I sourdough. Sourdough addition resulted in the pH of dough lower than 4.0, which is known to promote the solubility and degradation of gluten by cereal aspartic proteases, releasing elevated levels of, for instance, leucine, a precursor of isoamyl alcohol [2]. Yeast can convert free amino acids to fusel alcohols through the Ehrlich pathway [34]. As indicated in Figure 4B, our results show that leucine is converted into 3-methylbutanol by *S. cerevisiae* during proofing, which resulted in a lower concentration of leucine and a higher content of 3-methylbutanol in the proofed dough fermented with baker’s yeast comparing to the proofed dough fermented with type I sourdough. Similar to ethyl acetate, a reduction in 3-methylbutanol content was found in all CSBs that evaporated during steaming. Moreover, asparagine as the precursor of 2,3-butanedione is derived from homofermentative LAB fermentation and the Maillard reaction [35]. However, we did not find 2,3-butanedione in the final products after steaming possibly because of the use of *Lactobacillus sanfranciscensis* and other heterofermentative LABs as dominant microbiota of the type I sourdough starter cultures [7]. We found a high aldehyde content after proofing as well as after steaming. These results are consistent with the proposition that aldehydes can be generated from the oxidation of polyunsaturated fatty acids in the presence of lipoxygenase, which is present in flour. This gives the idea that the oxidation of polyunsaturated fatty acids by lipoxygenase is a phenomenon that happens during proofing and steaming [36]. As shown in Figure 4B, unsaturated fatty acids (palmitoleic acid and linoleic acid) in the proofed dough fermented with type I sourdough were lower as compared to the dough fermented by yeast. However, higher contents of hexanal, nonanal, (E)-2-octenal, (E)-2-nonenal, 2,4-decadienal and 2-pentyl furan were generated in the corresponding proofed dough, and their concentrations were further increased during steaming expect for nonanal. Liao et al. [37] reported that the generation of hydrogen peroxide by lactobacilli and *S. cerevisiae* can enhance lipid oxidation to promote the oxidation of linoleic acid leading to the synthesis of (E,E)-2,4-decadienal in wheat dough, which is the possible reason why lower concentrations of unsaturated fatty acids were found in type I sourdough-based proofed dough in this study. The potential generation mechanisms of these compounds were confirmed by metabolites analysis, which provided a theoretical basis for further research on the directional adjustment and improvement the aroma quality of CSB.

## 4. Materials and Methods 

### 4.1. Starter Collection and Flour Properties

Six type I sourdough samples were collected from Shandong Province in the northern part of China (TS1 to TS6); these are locally used to produce CSB with type I sourdough. Six different brands of baker’s yeast were purchased from local supermarkets in Wuxi (Y1 to Y6, Guangzhou Fuzheng Donghai Foods CO., LTD, ANGEL YEAST CO., LTD, AB Mauri UK & Ireland, NISSHIN SEIFUN OYC CO., LTD, Lesaffre Group and Heilongjiang Jiuding Yeast CO., LTD.). Moisture, ash and protein contents of the flour samples were also detected according to the approved AACCI methods 44-15A, 08-01 and 46-12, respectively [38].

### 4.2. Chinese Steamed Bread Samples Preparation

Batches, 500 g, of straight dough were processed by using a mixer (KM080, Kenwood, London, UK). It was assured that both types of doughs, containing either dry baker’s yeast or type I sourdough, had the same quantity of flour and moisture content (Table 3). After mixing, the dough was divided into 80 g/piece for rounding and proofing (40 min for yeast group and 60 min for type I sourdough group) at 35 °C and 80% relative humidity. The proofed buns were steamed for 20 min using a steaming oven (HB24D552W, SIEMENS, Berlin, Germany). Samples of each group were processed in duplicate.

### 4.3. Electronic Nose (E-Nose) Analysis

The volatile compounds of CSB were monitored by an e-nose (ISENSO INTELLIGENT., Shanghai China) according to the method described by Kachele [39] with some modifications. Each CSB sample (5 g) produced with either type I sourdough or active dry yeast was cut into small cubes and placed into 45 mL glass air tight vials with a screw cap. The sensor array sucked the gaseous compounds from the headspace of the samples at 400 mL/min for 180 s. After analysis, the system was purged for 150 s at a flow rate of 600 mL/min with filtered air before the next sample detection. The sensor array system contained 14 MOS of different chemical composition and thickness to provide selectivity towards volatile compounds. The records of individual MOS were used for principal component analysis (PCA).

### 4.4. SPME-GC/MS Analysis and Identification of Aroma Compounds

Volatile compounds of proofed dough and CSBs produced with type I sourdough and baker’s yeast were determined by SPME-GC/MS. The SPME device (Supelco, Bellefonte, PA, USA) was equipped with a 75 mm divinylbenzene/carboxen /polydimethylsiloxane and was operated as previously report [1]. Then, 3 g of each sample was cut into pieces and placed in a vial (20 mL) sealed with a screw cap. Subsequently, 5 μL methyl heptanoate (10 ppm) was added as an internal standard for relative concentration quantification; after 15 min of equilibration at 60 °C, the volatile compounds were absorbed by the fiber during a 30 min extraction. The GC was coupled to a Thermo Fisher Trace GC ultra-TSQ Quantum XLS mass selective detector. The column was TG-Wax (30 m × 0.25 mm, 0.25 μm film thickness) with 1.0 mL/min Helium as carrier gas. The GC (TSQ8000 Evo, Thermos, Waltham, MA, USA) temperature was held at 40 °C for 2 min, increased to 150 °C at a rate of 3 °C/min and then to 220 °C at a rate of 20 °C/min, and held for 1 min. The electron impact energy was 70 eV. The ion source and transfer line temperatures were set at 230 and 250 °C, respectively. The electron impact mass spectra ranged from 33 to 400. The identification of volatile compounds was performed by comparison of the mass spectral data obtained with those in the NIST library and Wiley Library, reporting the compounds with matching degree and purity above 800, and retention index (RI) using a C7-C30 *n*-alkanes series (Sigma-Aldrich, St. Louis, MO, USA) according to the method engaged by Cavalli [40].

### 4.5. Extraction and Derivatization of Proofed Dough for GC-TOFMS Analysis

In this analysis, 50 mg of each proofed dough sample was placed in the 2 mL microcentrifuge tube and extracted with 0.4 mL extraction liquid (Vmethanol:VH2O = 3: 1), followed by addition of 20 μL of ribitol (2 mg/mL stock in dH2O) as an internal standard. The mixture was further homogenized in a ball mill for 4 min at 40 Hz, then ultrasound treated for 5 min (incubated in ice water); and centrifuged for 15 min at 12,000 rpm, 4 °C. Then, 100 μL supernatant was transferred for drying by using a vacuum concentrator (RC1022, Thermos, Waltham, MA, USA). The derivatization of samples was carried as previously described by Zhao et al. [41]. 

GC-TOFMS analysis was performed using the Agilent 7890 gas chromatograph system coupled with a Pegasus HT time-of-flight mass spectrometer (Pegasus, St. Joseph, MI, USA). The system utilized a DB-5MS capillary column coated with 5% diphenyl cross-linked with 95% dimethylpolysiloxane (30 m × 250 μm inner diameter, 0.25 μm film thickness; J&W Scientific, Folsom, CA, USA). A 1 μL aliquot of the derivatized extract was injected in splitless mode. Helium was used as the carrier gas, the front inlet purge flow was 3 mL/min, while the gas flow rate through the column was 2.0 mL/min. The initial temperature was kept at 50 °C for 1 min, then raised to 320 °C at a rate of 10 °C/min, then kept for 5 min at 320 °C. The injection, transfer line, and ion source temperatures were 280, 280, and 220 °C, respectively. The energy was −70 eV in electron impact mode. The mass spectrometry data were acquired in full-scan mode with the m/z range of 85–600 at a rate of 20 spectra per second after a solvent delay of 366 s.

### 4.6. Metabolite Profiling

Shimadzu GCMS PostRun software was used to convert the raw data to “mzXML” format, then converted to “abf” format with the ABF converter. The MS DIAL equipped with FiehnLib was used for raw peak exaction, peak alignment, deconvolution analysis and identification [42,43]. Parameter settings were as follows: average peak width: 20; scan and minimum peak height: 10,000; sigma window value: 0.5, EI spectra cut off: 5000. The retention time tolerance: 0.5 min, the m/z tolerance: 0.5 Da, the EI similarity cut off: 70%, the identification score cut off: 70%. Alignment settings: retention time tolerance: 0.075 min, retention time factor: 0.5.

### 4.7. Multi-Statistical Analysis

The determination of each experimental procedure (E-nose, SPME-GC/MS and GC-TOFMS) were performed in triplicate. Values were expressed as mean values ± standard deviations. Significant differences among values were calculated based on Tukey’s procedure at *p* < 0.05, using the software SPSS (version 17). After normalization of the data, PCA, partial least-squares-discriminant analysis (PLS-DA) and heatmaps were performed using MetaboAnalyst (http://www.metaboanalyst.ca) to analyze dissimilarities among the samples in terms of volatile components. To validate models, a 7-fold validation was applied to the PLS-DA model and the reliabilities of the models were further rigorously validated by a permutation test (*n* = 100) [41].

## 5. Conclusions

This study has provided a comprehensive overview of the key aroma compounds of CSB produced with type I sourdough through the untargeted metabolomics analysis and odor threshold techniques. As a result of using these two methods, we concluded that ethyl acetate (fruity), ethyl lactate (caramel-like), hexyl acetate (fruity), (E)-2-nonenal (fatty) and 2-pentylfuran (fruity), having higher VIP and OAV values, are the key aroma compounds in type I sourdough-based steamed breads as compared to the breads fermented with baker’s yeast. The metabolite analysis of the proofed dough further revealed precursor compounds that were generated during the proofing process and later transformed into steamed breads after the steaming process.

## Figures and Tables

**Table 1:** Aroma discriminant compounds in Chinese steamed bread produced with type I sourdough and baker’s yeast and their odor activity values, determined using PLS-DA.

Discriminant Marker	Group	Trend ^a^	VIP ^b^	Odor Threshold ^c^	OAV ^d^	
					Y	TS
Acetic acid	acid	Up	1.23	22,000	<1	<1
2-Methyl-1-propanol	alcohol	Up	1.23	7000	<1	<1
Ethanol		Up	1.21	100,000	<1	<1
1-Pentanol		Up	1.01	4000	<1	<1
Ethyl lactate	ester	Up	1.24	10	<1	6.7
Ethyl acetate		Up	1.22	5	<1	36.2
Ethyl octanoate		Up	1.21	-	-	-
Hexyl acetate		Up	1.14	2	<1	7.1
(E)-2-Nonenal	aldehyde	Up	1.10	0.08–0.1	45.4–56.7	111–139
Pentanal		Up	1.08	12–42	<1	<1
(E)-2-Octenal		Up	1.08	3	1.2	2.3
Hexanal		Up	1.01	4.5–5	2.9–3.2	5.0–5.4
2-Pentyl furan	furan	Up	1.14	6	14.6	43.2

^a^ The trend (UP or Down) relates to their concentration in CSBs with type I sourdough as compared to the those produced with baker’s yeast. ^b^ Variable importance in the projection values. ^c^ Threshold values in water measured following the method of according to Leffingwell et al. (2016) (ppb). ^d^ The odor activity values (OAV) were calculated by dividing the concentrations of the odorants by their nasal odor detection thresholds.

**Table 2:** Selected discriminant precursors in proofed dough fermented with type I sourdough as compared to those proofed with baker’s yeast.

No.	Discriminant Marker	Group	RT (time)	Trend ^a^	VIP ^b^
1	4-Hydroxybutyric acid	Acids	10.00	Up	2.23
2	Malic acid		13.42	Up	2.20
3	Citric acid		17.19	Up	2.17
4	Lactic acid		7.48	Up	1.53
5	3-Phenyllactic acid		14.58	Up	1.45
6	Leucine	Amino acids	10.53	Up	1.68
7	Histidine		18.32	Up	1.62
8	Asparagine		15.57	Up	1.43
9	Glutamic acid		14.99	Up	1.12
10	Palmitoleic acid	Fatty acids	19.31	Down	1.54
11	Linoleic acid		21.04	Down	1.20

^a^ The trend (Up or Down) relates to their concentration in proofed dough with type I sourdough as compared to the those proofed with baker’s yeast. ^b^ Variable importance in the projection values.

**Table 3:** Composition of straight dough produced with baker’s yeast and type I sourdough.

	Wheat Flour/g	Water/g	Yeast/g	Sourdough/g
Y group	340	160	5.1	/
TS group	275	125	/	100

## Supplementary Materials

Supplementary materials can be found at http://www.mdpi.com/1422-0067/20/4/818/s1.

## Abbreviations

CSB	Chinese steamed bread
OAVs	Odor activity values
MOS	Metal oxide semiconductors
PCA	Principal component analysis
SPME	Solid-phase microextraction
GC/MS	Gas chromatography coupled to mass spectrometry
RI	Retention index
NIST	National Institute of Standards and Technology
GC-TOFMS	Gas chromatography / Time-of-flight mass spectrometers
PLS-DA	Partial least-squares-discriminant analysis
LAB	Lactic acid bacteria
VIP	Variable important in the projection
