# Supplementary material for: Identification of Key Aroma Compounds in Type I Sourdough-Based Chinese Steamed Bread: Application of Untargeted Metabolomics Analysisp
Source: Int J Mol Sci. 2019 Feb 14;20(4):818. doi: 10.3390/ijms20040818 (PMC6412449; doi:10.3390/ijms20040818)
Supplement: Supplementary file 1 [file ijms-20-00818-s001.pdf]

Supplementary data

Table.S1 Identification and quantification of volatile compounds of proofed dough and CSBs produced with type I sourdough and baker's yeast

ID	Volatiles compound	Calculated RI	RI as reporteda	After steaming (ug/kg)		After proofing (ug/kg)	
				Y	TS	YD	TSD
1	Ethyl Acetate	890.751	890	1.55±1.32	181±83	32.5±4.4	244±127
2	3-hydroxy Butanal	914.451	—	0.38±0.10	0.63±0.43	0.54±0.10	0.24±0.17
3	Ethanol	930.058	930	412±36	1234±236	420±36	413±156
4	Pentanal	977.456	978	1.02±0.24	2.29±0.61	0.34±0.09	0.88±0.37
5	Butanoic acid methyl ester	984.971	—	1.72±0.14	1.17±0.36	0.60±0.05	0.48±0.13
6	Hexanal	1081.091	1081	14.3±1.78	24.9±7.64	4.14±1.28	17.2±7.0
7	2-methyl-1-Propanol	1092.364	1093	0.03±0.01	1.67±0.75	17.6±1.6	2.36±2.15
8	3-methyl-1-Butanol	1208.416	1206	67±11	9.15±8.48	82.0±16.6	22.7±8.3
9	2-pentyl-Furan	1230.693	1231	87.7±12.7	259±121	1.34±0.54	4.81±1.72
10	1-Pentanol	1251.485	1252	3.56±0.24	6.51±3.30	2.15±0.55	4.14±1.68
11	5-methyl-3-Heptantone	1253.713	—	1.44±0.12	1.44±0.67	0.47±0.17	0.16±0.11
12	Hexyl acetate	1273.762	1275	1.13±0.79	14.2±8.2	1.05±0.10	2.65±0.90
13	2-Octanone	1284.653	1283	3.57±0.37	1.67±0.73	2.15±0.40	0.40±0.26
14	6-methyl-5-Hepten-2-one	1337.981	1339	3.26±0.38	3.89±1.12	1.70±0.36	0.87±0.36
15	Ethyl lactate	1344.952	1341	0.29±0.08	67.1±24.0	0.02±0.01	23.1±13.1
16	1-Hexanol	1355.048	1353	92.4±8.0	114±54	54.5±7.1	52.8±23.2
17	Nonanal	1393.99	1394	34.6±8.7	7.51±7.09	4.18±1.88	32.2±17.8
18	trans-2-Undecen-1-ol	1401.707	—	0.33±0.05	1.03±0.25	0.25±0.06	0.30±0.12
19	(E)-2-Octenal	1428.78	1425	3.64±0.46	6.99±2.58	0.91±0.12	5.32±3.28
20	Ethyl octanoate	1436.098	—	3.09±0.47	10.3±4.5	0.50±0.10	5.32±3.28
21	1-Octen-3-ol	1453.415	1445	33.4±2.9	40.7±14.1	10.6±1.7	20.1±7.9
22	Heptanol	1457.561	1457	8.89±1.75	6.63±1.86	5.05±1.18	3.61±0.83
23	Acetic acid	1465.609	1459	0.24±0.07	85.4±37.3	0.06±0.01	21.6±4.5
24	Benzaldehyde	1521.717	1522	13.3±4.5	11.6±2.5	1.99±0.61	1.12±0.82
25	(E)-2-Nonenal	1532.683	1532	4.54±1.03	11.1±3.1	1.28±0.28	2.12±0.71
26	1-Octanol	1559.596	1565	5.00±1.18	3.66±1.32	4.27±1.51	3.29±1.17
27	2-Octen-1-ol	1614.286	—	0.47±0.11	6.87±6.09	0.27±0.12	6.05±4.46
28	1-Nonanol	1661.417	1671	3.09±0.71	4.84±2.18	4.82±1.25	4.15±1.18
29	3-Nonen-1-ol	1683.990	—	7.54±2.23	4.12±1.29	4.80±1.50	2.55±0.83
30	Z-2-Dodecenol	1715.068	—	0.60±0.06	2.16±0.98	0.37±0.10	1.81±1.03
31	Naphthalene	1731.233	1740	4.75±0.89	27.9±23.8	1.52±0.56	4.69±4.16
32	2,4-Decadienal	1810.042	1816	2.32±0.23	3.04±1.21	1.25±0.52	5.94±2.46
33	Ethyl phenylacetate	1816.318	—	3.76±0.64	3.15±2.02	0.91±0.40	2.73±1.77
34	Phenylethyl Alcohol	1907.241	1903	116±19	12.8±11.2	38.7±11.2	33.0±21.8

a RI Values as reported in http://www.odour.org.uk.

Table.S2 Candidates discriminant compounds in proofed dough fermented with type I sourdough as compared to those proofed with baker's yeast

	Discriminant marker	RT (time)	Trend a	VIP b
1	4-hydroxybutyric acid	10.00	up	2.22964
2	malic acid	13.42	up	2.19594
3	citric acid	17.19	up	2.17217
4	Phosphate	10.56	up	2.17113
5	Malate	13.40	down	1.78963
6	Xylulose	15.62	up	1.77082
7	Ribose	15.63	up	1.76077
8	Phenol	7.42	down	1.71371
9	Leucine	10.53	up	1.68524
10	Histidine	18.32	up	1.62382
11	dehydroascorbic acid	17.49	down	1.58808
12	myristic acid	17.56	down	1.55806
13	beta-gentiobiose	26.11	down	1.54878
14	lactic acid	7.48	up	1.52917
15	palmitoleic acid	19.31	up	1.5144
16	Galactinol	26.94	down	1.49616
17	2-deoxyerythritol	10.78	down	1.46825
18	3-phenyllactic acid	14.58	up	1.45479
19	alpha-ketoglutarate	14.44	up	1.44564
20	Asparagine	15.57	up	1.42793
21	Lyxose	15.28	up	1.37216
22	maleic acid	10.97	down	1.3165
23	Palatinitol	26.43	up	1.30598
24	fumaric acid	11.62	down	1.28641
25	glucose-1-phosphate	16.64	up	1.22388
26	Xylose	15.46	up	1.2012
27	3-hydroxypropionic acid	8.70	down	1.20088
28	linoleic acid	21.04	down	1.19952
29	Diglycerol	16.17	down	1.19778
30	isothreonic acid	14.25	down	1.19159
31	Sucrose	24.47	up	1.1835
32	Sophorose	25.65	up	1.18126
33	glutamic acid	14.99	up	1.122
34	2-monopalmitin	23.85	up	1.11968
35	diacetone alcohol	7.24	down	1.11388
36	butane-2,3-diol	7.06	down	1.08526
37	Threitol	13.62	up	1.07723
38	Maleimide	7.92	up	1.02595
39	nicotinic acid	10.91	down	1.02047
40	Oxoproline	13.84	down	1.01942

41	glyceric acid	11.31	down	1.00637

a The trend (UP or Down) relates to their concentration in proofed dough with type I sourdough as compared to the those proofed with baker's yeast.

b: variable importance in the projection values

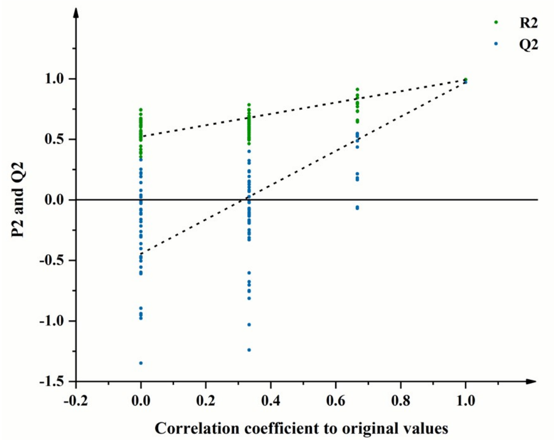

Figure.S1 Permutation test of partial least-squares-discriminant analysis (PLS-DA) models of the headspace volatiles identified in Chinese steamed bread produced with baker's yeast (Y) and type I sourdough (TS)

(A) (B)

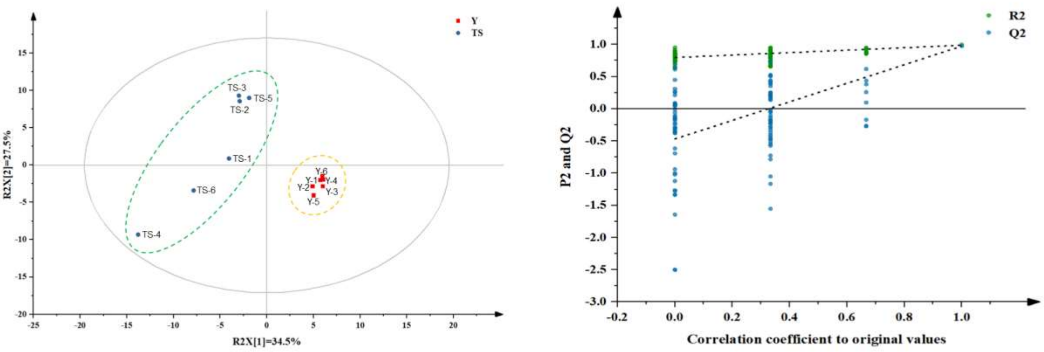

Figure.S2 Principal component analysis (PCA) scores plot (A) and permutation test of partial least-squares-discriminant analysis (PLS-DA) models (B) of the potential metabolite markers in proofed dough fermented with type I sourdough (TS) and baker's yeast (Y)

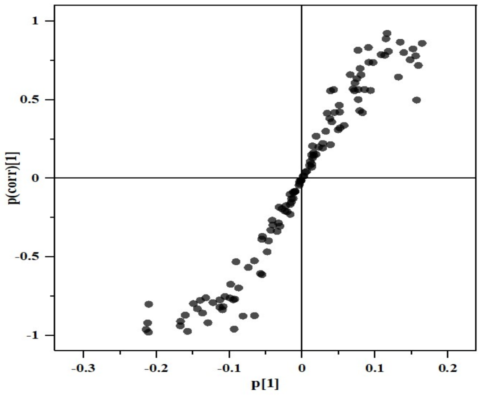

Fig.S3 S-plot of the potential metabolite markers in proofed dough fermented with type I sourdough (TS) and baker's yeast (Y)
